# Impact of blood flow restriction cuff design on upper body exercise: A randomized crossover trial in resistance‐trained adults

**DOI:** 10.14814/phy2.70303

**Published:** 2025-04-07

**Authors:** Nicholas Rolnick, Victor S. de Queiros, Brent Fedorko, Samantha Watson, Campbell Ruffhead, Sean Zupnik, Lucas Kuriawa, Mark Weedon, Tim Werner

**Affiliations:** ^1^ Department of Exercise Science and Recreation CUNY Lehman College New York New York USA; ^2^ The BFR PROS New York New York USA; ^3^ Department of Physical Education State University of Paraíba (UEPB) Campina Grande Paraíba Brazil; ^4^ Department of Exercise Science Salisbury University Salisbury Maryland USA

**Keywords:** arterial stiffness, B strong, Delfi, exercise performance, perceptual responses, resistance training

## Abstract

Differences in cuff blood flow restriction (BFR) bladder design (single‐chambered [SC‐BFR] and multi‐chambered [MC‐BFR] systems) may influence exercise performance, perceptual responses, and cardiovascular outcomes. In a randomized cross‐over design, twenty‐six healthy physically active individuals (22.6 ± 5.5 years old, 10 females; 25 reported engaging in resistance‐exercise consistently) performed four sets of bilateral biceps curls to volitional failure using 20% of the 1‐repetition maximum under three conditions: SC‐BFR, MC‐BFR, and a non‐BFR control, post‐exercise perceptual responses, and cardiovascular measures pre‐ and post‐exercise. SC‐BFR significantly reduced total repetitions compared to MC‐BFR and N‐BFR (*p* < 0.001). MC‐BFR and N‐BFR conditions demonstrated comparable performance in later sets. RPD was significantly higher in SC‐BFR compared to MC‐BFR and N‐BFR (*p* < 0.001), while MC‐BFR elicited lower RPE than SC‐BFR (*p* = 0.025). Both SC‐BFR and N‐BFR conditions significantly reduced post‐exercise diastolic blood pressure and mean arterial pressure, whereas MC‐BFR did not. No significant differences in PWV were observed across conditions. SC‐BFR induces greater repetition reduction and perceptual discomfort than MC‐BFR, while MC‐BFR demonstrates similar performances and comfort to N‐BFR in later sets. Findings suggest cuff design plays a role in acute BFR responses.

## 1 INTRODUCTION

Blood flow restriction (BFR) training typically involves using pneumatic devices that can adjust pressure to an individual's specific limb occlusion pressure (LOP) (Scott et al., 2023). Studies suggest that applying pressures at or above 50% of LOP significantly accelerates muscle fatigue during resistance exercises (shown by a decrease in repetitions to volitional muscular failure), likely due to increased local metabolic stress (Cerqueira et al., 2021). Consequently, BFR pressure levels between 40% and 80% of LOP, with loads ranging from 20% to 30% of one‐repetition maximum, may have practical significance in optimizing results, particularly for those that are load and/or pain compromised, preventing them from lifting heavier weights (Jacobs et al., 2025; Noyes et al., 2021).

The expansion of BFR across various practice settings (Scott et al., 2023) has led to the development of numerous cuffs with varying features and characteristics (Rolnick et al., 2023). Among these features, the air bladder system design has drawn some attention in research, albeit with some limitations (Rolnick, 2024). Conventional medical tourniquets, which are widely used and capable of individualized pressure adjustment, typically employ single‐chambered air bladders with wider cuffs (Graham et al., 1993). This design distributes pressure circumferentially around the limb, which can reduce or even restrict arterial inflow and venous outflow based on the applied pressure. These single‐chambered systems are the most used in practice and are well documented in both surgical and BFR research (Patterson et al., 2019).

Multi‐chambered bladder systems are designed with multiple sequential bladders separated by small gaps, leaving portions of the limb without direct compression when inflated fully (Rolnick et al., 2023). This structure makes achieving individualized occlusion pressures challenging. As a result, high set pressures (e.g., pressure the user sets) may reduce venous return without adequately restricting arterial inflow, making personalized pressure application difficult. Consequently, such devices typically recommend starting pressures of 200 mmHg for upper limbs and 300 mmHg for lower limbs as a general guideline (Early et al., 2020).

The single‐ versus multi‐chambered bladder designs may influence exercise performance and perceptual outcomes (Dancy et al., 2023; Rolnick, de Queiros, Moghaddam, Peikon, et al., 2024). One study that implemented bilateral lower body exercise in multiple sets to volitional muscular failure suggests that single‐chambered cuffs (e.g., Delfi Personalized Tourniquet [Delfi, Vancouver, Canada; 11.5 cm cuff width]) lead to greater reductions in median total repetition counts compared to multi‐chambered systems (e.g., B Strong [B Strong, Park City, UT; 7.5 cm cuff width]) and no‐BFR conditions (Rolnick, de Queiros, Moghaddam, Peikon, et al., 2024). Participants using single‐chambered cuffs also showed significant drops in repetitions to volitional failure compared to no‐BFR during sets 2–4, with no differences observed in the multi‐chambered cuffs. However, both cuffs led to elevated perceived discomfort levels, possibly due to the impact of BFR during strenuous resistance exercise. Nonetheless, the finding of enhanced fatiguability in single‐chambered cuffs is not a universal finding. Dancy et al. (2023) implemented two sets of elbow flexion exercise to volitional failure in healthy adults using two single‐chambered BFR cuffs—the Delfi Personalized Tourniquet, SmartCuffs [SmartTools, Ohio; 6.3 cm cuff width] both inflated to 50% arterial occlusion pressure‐ and one multi‐chambered BFR cuff—B Strong inflated to 200 mmHg as per the manufacturer's recommendation of starting pressures. The results indicate no difference in performance, perceptual experiences, or acute safety measures between devices. However, important limitations warrant caution when extrapolating these findings to practice. First, participants performed exercises with only two cuff conditions instead of three. Second, the exercise protocol consisted of only two sets, which does not reflect typical practice (Patterson et al., 2019; Scott et al., 2023). Additionally, the Delfi Personalized Tourniquet Device significantly reduced repetitions and produced higher ratings of perceived exertion in the second set compared to the other cuff conditions. The study's two‐set design may have missed effects that could emerge in a more common four‐set routine, as shown in Rolnick, de Queiros, Moghaddam, Peikon, et al. (2024). It is unknown whether the impact of bladder design is only relevant in lower body exercise, or if the effect was missed by Dancy et al. (2023)'s upper body two‐set design.

Arterial stiffness represents a pathological adaptation due to mechanical stressors, leading to collagen fiber buildup and a gradual loss of elastic proteins (Mitchell, 2009; Zieman et al., 2005) in the tunica media and adventitia layers. It is commonly measured in the aorta, brachial, and femoral arteries, with pulse wave velocity (PWV) serving as the gold‐standard assessment method (Laurent et al., 2006). PWV assesses the speed at which blood pressure waves move through the arteries, with higher speeds indicating stiffer, less elastic arteries (Reference Values for Arterial Stiffness' Collaboration, 2010). Chronic increases in PWV are an independent risk factor for cardiovascular events including myocardial infarction and stroke (Laurent et al., 2006; London et al., 2004). However, there is limited research on the acute PWV response during resistance exercise with BFR and on whether this response varies depending on the design of the BFR cuff. Currently, only one study exists that assessed arterial stiffness responses following lower body exercise (Rolnick, de Queiros, Moghaddam, Peikon, et al., 2024) and determined that there may be an effect of blunting post‐exercise stiffness using single‐chambered BFR devices, but not multi‐chambered devices or low‐intensity exercise without BFR. There is an absence of evidence on the impact of upper body exercise on post‐exercise arterial stiffness responses.

Therefore, the purpose of this study was to compare a single‐ (Delfi Personalized Tourniquet Device) (SC‐BFR) versus multi‐chambered (B Strong) (MC‐BFR) cuff design on acute performance, perceptual, and cardiovascular (e.g., peripheral and central measures of arterial stiffness) outcome measures during 4 sets of upper body bilateral biceps curl exercise to volitional failure using recommended application BFR guidelines (Patterson et al., 2019). We hypothesized that the SC‐BFR would display the largest reductions in repetitions to fatigue, followed by the MC‐BFR, which would display a greater reduction in repetitions to fatigue compared to free‐flow exercise (N‐BFR) performed at the same intensity. In addition, we hypothesized that perceptual experiences would be heightened in the SC‐BFR and attenuated in the MC‐BFR condition, but both elevated above N‐BFR. Last, we hypothesized that there would be no impact of exercise performance on acute arterial stiffness responses, as shown in prior research performing a similar repetition protocol in the upper body. This study aims to shed light on how different cuff designs may impact training outcomes and safety, offering practical guidance for their effective application in resistance exercise with blood flow restriction.

## 2 MATERIALS AND METHODS

### 2.1 Participants

G*Power (Kiel University, Germany) software 3.1.9.7 calculated a sample size of 24 for three study groups using pre‐ and post‐measurements and repeated measures analysis of variance (ANOVA) at *α* = 0.05 & *β* = 0.80 to detect partial eta‐squared (*η* ^2^ *p*) = 0.11. To account for attrition, 28 participants 18–40 years of age of all races and ethnic backgrounds meeting the inclusion and exclusion criteria were initially recruited for the study. Inclusion criteria included 18–40 years of age, weight stable (less than 2.5 kg weight fluctuation in the previous 6 months), and all female participants were eumenorrheic for at least the last 2 years. Menstrual patterns were not controlled for due to the nature of the randomization process. Additionally, several recent randomized controlled trials and systematic reviews have called into question the impact of menstrual cycles on vascular compliance (Priest et al., 2018; Shenouda et al., 2018; Williams et al., 2020, 2024). To qualify for the study, participants also must have met the minimal guidelines for physical activity in the previous 6 months (American College of Sports Medicine et al., 2022). Exclusion criteria were a diagnosis of diabetes, cardiovascular, liver, and/or kidney disease; stage 2 hypertension, sleep apnea, morbid obesity, acute surgery (<2 months before data collection), and current or past use of tobacco products (Nascimento et al., 2022). Each participant signed an informed consent document in accordance with the Declaration of Helsinki acknowledging potential risks and harms. The study was approved by the ethics committee of Salisbury University (protocol #364) and registered at clinicaltrials.gov (NCT06754618).

### 2.2 Experimental design

**FIGURE 1:**
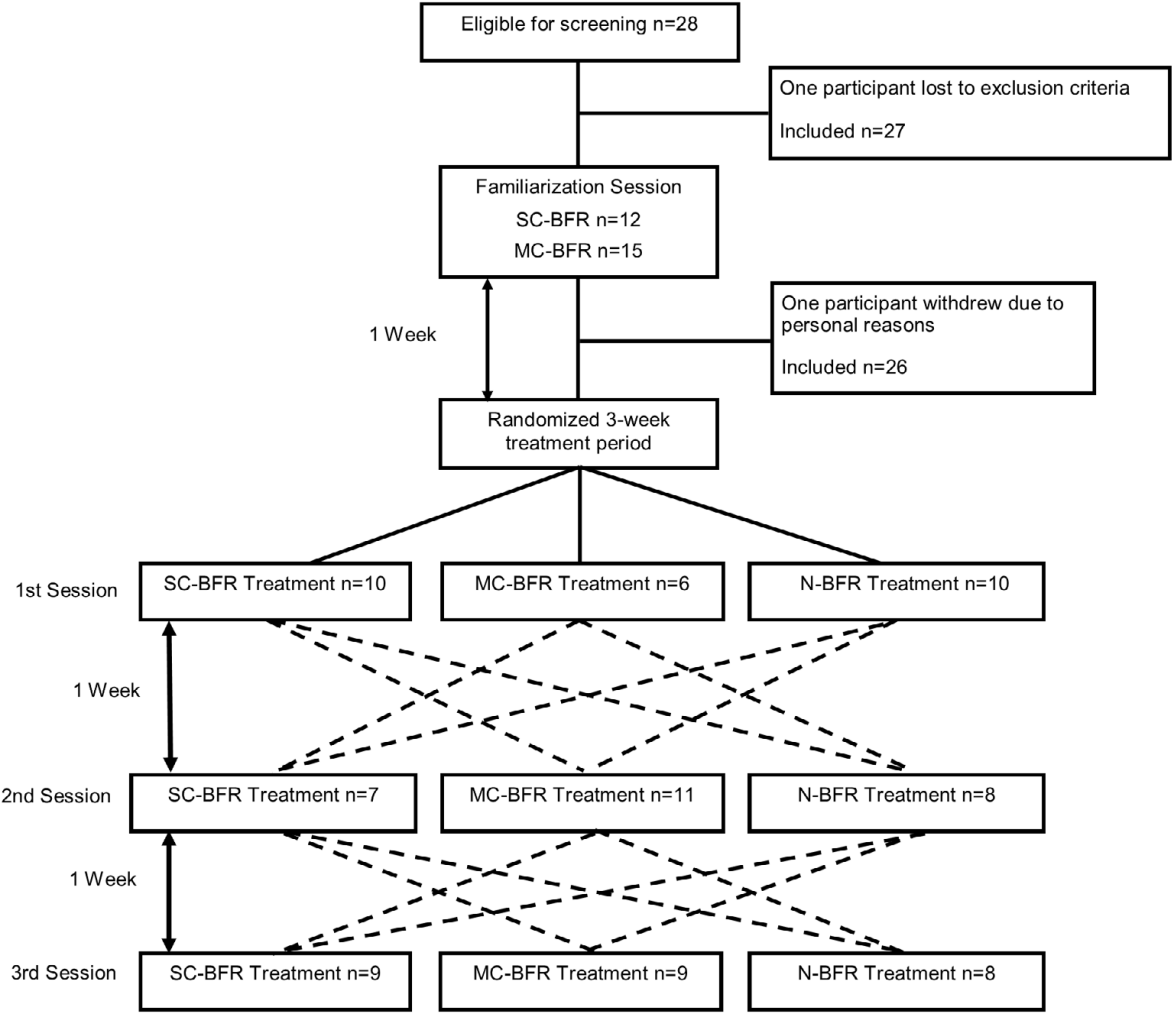
Experimental design of the trial. All participants performed a familiarization session followed by three randomized experimental sessions. MC‐BFR, multi‐chambered BFR cuff; N‐BFR, no BFR; SC‐BFR, single‐chambered BFR cuff.

The purpose of this study was to investigate the effects of exhaustive low‐load upper body resistance exercise with and without blood flow restriction (BFR) on acute performance, cardiovascular, and perceptual responses. The experimental trials involved performing low‐load bilateral biceps curls to volitional failure using three different conditions: SC‐BFR cuffs, MC‐BFR, or N‐BFR. This study employed a randomized, crossover design, with session order determined using randomization software (www.random.org). Participants visited the lab on four separate occasions (Figure 1). The first visit included a familiarization session and an exercise training regimen using the BFR cuffs. During the subsequent sessions, participants completed each of the three treatment conditions (SC‐BFR, MC‐BFR, N‐BFR) in a randomized order. Each session, including the familiarization, was separated by a one‐week washout period, scheduled at the same time of day to minimize the effects of prior exposure, allow recovery, and limit diurnal influences. Additionally, all participants were instructed to maintain consistent physical activity levels throughout the experiment to minimize the impact of activity changes on study outcomes.

### 2.3 Procedures

Exercise and data collection took place in the Exercise Physiology Research Laboratory between 07:00 and 13:00 h on the same day each week with temperatures in the lab controlled between 21°C and 23°C. Participants were instructed to maintain their usual exercise and dietary routines throughout the study and to refrain from caffeine, alcohol, and exercise for 24 h before each session. Additionally, participants reported fasting for at least 4 h before the start of each session.

Each participant was initially assessed for their one repetition maximum (1‐RM) according to ACSM guidelines (American College of Sports Medicine et al., 2022). Only one 1‐RM test was conducted during the familiarization period, as the primary focus was on comparing acute physiological and perceptual responses across conditions rather than longitudinal training effects. During the familiarization and subsequent SC‐BFR, MC‐BFR, and N‐BFR sessions, participants performed four sets of dumbbell biceps curls to volitional fatigue using 20% of their 1‐RM, rounded to the nearest 5 lb. increment.

In each session, participants held a dumbbell in each hand, standing with shoulders flexed at 0°, elbows fully extended (~180°) in supination. Each repetition began with an upward (concentric) rotation of the elbow until limited by anatomy or the BFR cuff, followed by a downward (eccentric) return to the starting position. A metronome (Seiko, Mahwah, NJ) controlled the cadence, set to 2 s for each concentric and eccentric phase, with a 1‐min inter‐set rest. This cadence and rest period were chosen based on recent practice recommendations (Patterson et al., 2019). Volitional muscular failure was defined as the participant's inability to maintain cadence or form (e.g., incomplete elbow range, using unsanctioned movements like hip thrust), or voluntary termination. Participants received verbal warnings on the first form violation, with a subsequent violation ending the set.

**FIGURE 2:**
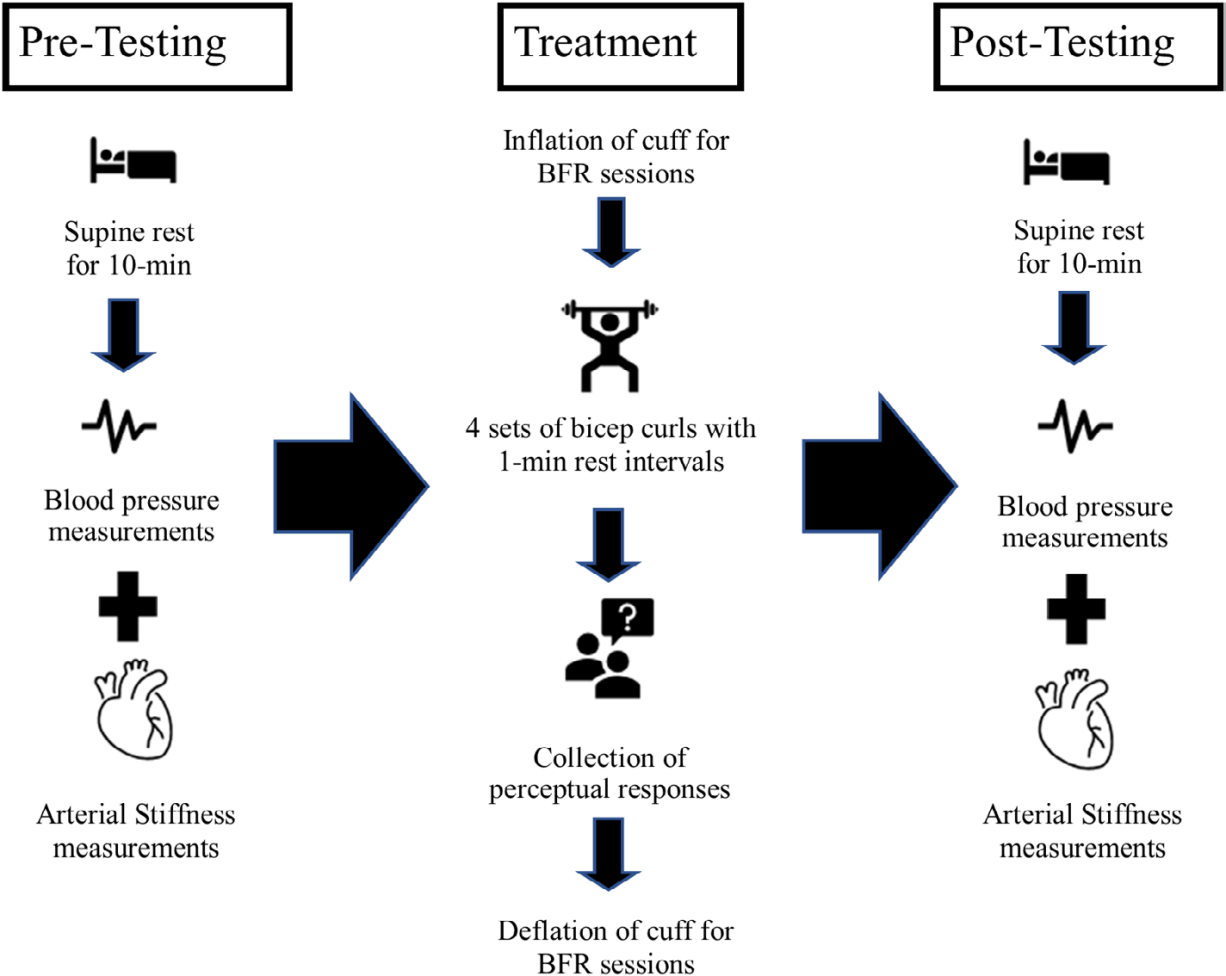
Session overview. During each session, baseline cardiovascular assessments were performed followed by the randomized allocation to an experimental arm.

Arterial stiffness was measured within 10 min before and after each session. Perceptual responses, including rating of perceived exertion (RPE) (0–10 rating scale), rating of perceived discomfort (RPD) (0–10 rating scale), and participant enjoyment (Likert 1–10 scale), were recorded immediately post‐exercise with the cuffs inflated to capture peak perceptual demands and enjoyment (Figure 2). Adverse events were monitored per established guidelines (Minniti et al., 2020).

Performance was evaluated by total repetitions and per‐set repetition volume changes across multiple sets between conditions. Total cuff inflation time was also tracked for both BFR conditions to assess potential differences in time exposure due to bladder design.

### 2.4 Anthropometrics and body composition

Anthropometric data were collected during the familiarization session. Body height was measured using a stadiometer (Detecto 439 Physician Beam Scale) with participants standing upright and barefoot. Total body mass, fat mass, and fat‐free mass were assessed using air displacement plethysmography (BOD POD) (Cosmed Metabolic Company, Rome). For this assessment, participants wore tight‐fitting clothing, were fully voided, and reported fasting for at least 4 h. All measurements were conducted in accordance with the manufacturer's specifications.

### 2.5 Brachial blood pressure

Following a 5–10 min rest in a supine position, brachial blood pressure (BP) readings were taken every 2 min in the right arm using an automated device (Welch Allyn, New York). All BP measurements followed American Heart Association guidelines (Whelton et al., 2018). Steady‐state BP was determined by averaging three consecutive readings within ±6 mmHg for both systolic (SBP) and diastolic (DBP) pressure. If steady state was not reached after six measurements, the last three readings were averaged for the final calculation.

### 2.6 Central pressures, pulse pressure, and pulse wave velocity

Central blood pressure (cSBP and cDBP) was determined using a device‐specific algorithm that matched brachial SBP and DBP with carotid pressure waveforms. Pulse pressure (PP) was determined by subtracting cDBP from cSBP. Pulse wave velocity (PWV) acquisition was conducted by the same examiner with an intraclass correlation coefficient (ICC) of 0.93 for PWV. All measurements adhered to manufacturer specifications and current guidelines, with assessments approximately 10 min post‐exercise (Boutouyrie et al., 2014; Laurent et al., 2006). After a minimum of 10 min resting in a supine position, arterial tonometers (Complior Analytic Tonometer, Alam Medical, Vincennes, France) were applied simultaneously to the right carotid, radial, and femoral arteries to calculate central (carotid‐femoral, cf) and peripheral (carotid‐radial, cr) PWV. The distance between arterial sites was measured using an extended caliper to the nearest 10 mm. PWV was calculated using the formula PWV = D (cm)/Δt (sec) after 10 cardiac cycles met the software's inclusion criteria. Perceptual Responses and Performance.

Immediately after the final set, while the cuffs remained inflated, participants responded to three assessments: rating of perceived exertion (RPE), rating of perceived discomfort (RPD), and a 1–10 Likert scale gauging the likelihood of performing the exercise again. A chart with response options was held at eye level, and questions were presented in the same order: “How hard were you working out?” “How much discomfort did you feel?” and “On a scale of 1–10, how likely would you be to perform this exercise again, with 10 being very likely and 1 being not likely at all?” Due to the timing of these questions, we anticipated capturing peak perceptual experiences. Additionally, repetitions per set and total repetitions across the four sets were recorded for each session.

### 2.7 Familiarization session

The first measurements recorded were seated blood pressures, adhering to current protocols (Whelton et al., 2018). Height, weight, and body composition were measured next. Participants then received instructions on recording perceptual responses, as outlined in previous interventions (Rolnick, de Queiros, Moghaddam, Peikon, et al., 2024; Rolnick, Licameli, Moghaddam, Marquette, et al., 2024). Following these steps, a one repetition maximum (1‐RM) bilateral dumbbell biceps curl test was conducted according to standardized guidelines (American College of Sports Medicine et al., 2022). The 1‐RM test was performed only once during the trial to set the load for the subsequent three sessions. Afterward, participants completed a randomized BFR training session using either the SC‐BFR or MC‐BFR cuffs, following the exercise protocol previously described (Rolnick, de Queiros, Moghaddam, Peikon, et al., 2024). No post‐exercise measurements were taken during the familiarization session.

### 2.8 BFR devices

**FIGURE 3:**
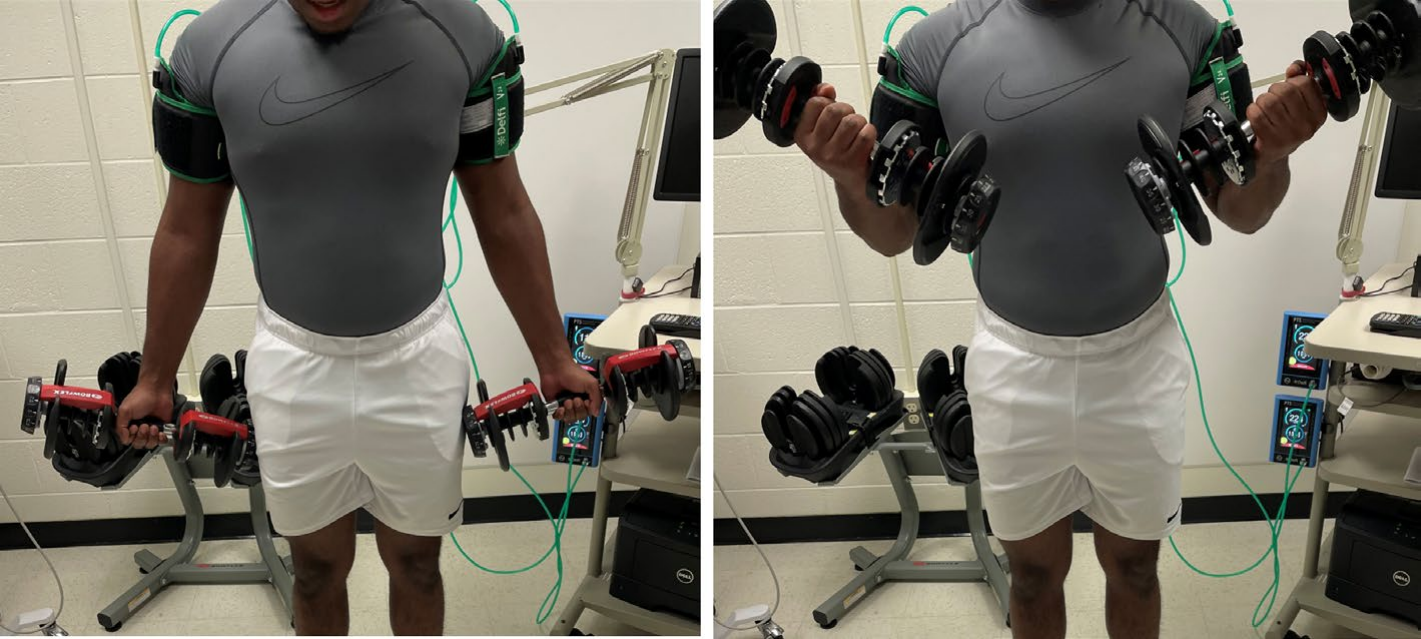
Biceps curl demonstration. This depicts the standard range of motion used during the bilateral biceps curl exercise employed in the current study.

**FIGURE 4:**
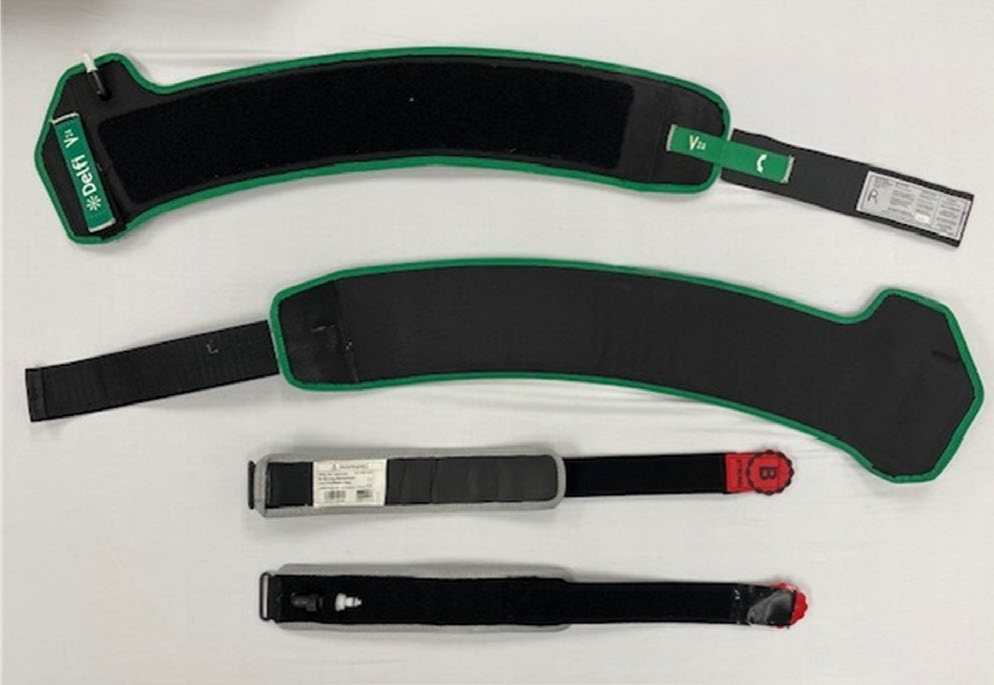
The BFR cuffs used during the trial. SC‐BFR is the Delfi Personalized Tourniquet Device and MC‐BFR is the B Strong Cuff.

The SC‐BFR (Delfi, Vancouver, Canada; 11.5 cm cuff width) and MC‐BFR (B‐Strong, Park City, UT; 7.5 cm cuff width) devices were used during two of the three exercise sessions (Figures 3 and 4). Cuffs were placed at the most proximal part of the right and left upper arms while participants were in a supine position. For the SC‐BFR device, limb occlusion pressure (LOP) was determined in the supine position per manufacturer specifications, and cuff pressure was set at 60% of LOP throughout the session. For the MC‐BFR device, cuff pressure was set to 200 mmHg in the supine position, using the red bands designated for upper body exercises, as recommended by the manufacturer. As MC‐BFR cuffs are marketed to be unable to occlude by the manufacturer, relativizing the applied pressure by standardizing according to LOP is not possible even with cuff pressures exceeding 500 mmHg (Safety, 2024). Therefore, we chose to investigate the recommended starting pressures according to the manufacturer and compare them to a moderate level of personalized pressure to increase external validity, as these pressure settings are often used in practice (Cuffe et al., 2022; Scott et al., 2023). Cuff pressure was maintained continuously during both exercise and rest periods for both BFR conditions and was only released when perceptual responses were recorded after the final set. Due to the study design, participants were not blinded to the treatment conditions. A control session without BFR cuffs (N‐BFR), following the same exercise protocol and intensity, was also performed in a randomized order.

### 2.9 Statistical analysis

Initially, data normality was assessed using the Shapiro–Wilk test. Due to the non‐normal distribution observed in the perceptual variables, Friedman's ANOVA was employed to evaluate the effect of the exercise sessions on RPE, RPD, and the likelihood of performing the exercise again. These results are presented descriptively as medians with interquartile ranges (25th and 75th percentiles). To analyze the effects of condition and time on blood pressure measures and pulse pressure, a two‐way repeated measures ANOVA was conducted with a [3] conditions × [2] times design. For other variables, the generalized estimating equation (GEE) with a Gamma distribution model was used to evaluate the effects of time, condition, and their interaction. Bonferroni's post hoc test was applied for pairwise comparisons to identify specific differences. The significance level was set at *p* < 0.05 for all analyses. Effect sizes were reported as η2p and were defined as: 0.01, small; 0.06, medium; and ≥0.14, large (Lachenbruch & Cohen, 1989). Data analysis was performed using IBM® SPSS Statistics software (version 24.0).

## 3 RESULTS

### 3.1 Participants

Participants' baseline characteristics are shown in Table 1. Twenty‐eight participants were screened for the study. One participant did not meet the inclusion/exclusion criteria. The other withdrew due to scheduling conflicts. Accordantly, 26 physically healthy and active participants (10 females) met the inclusion criteria volunteered for the study. The cohort's self‐identified racial makeup was 65% Caucasian, 19% African–American, and 15% Asian–American. Approximately 97% of the participants (*n* = 25) reported having more than 6 months of resistance training experience (Table 1).

**TABLE 1:** Participant characteristics.

Variable	Mean ± SD
Age, yr	22.6 ± 5.5
Height, cm	173.9 ± 10.97
Weight, kg	79.3 ± 19.0
BMI, kg/m^2^	26.1 ± 5.4
Body Fat, %	16.8 ± 11.3
Fat Mass, kg	14.3 ± 12.2
Fat Free Mass, kg	65.0 ± 13.8
Seated SBP, mmHg	122 ± 11
Seated DBP, mmHg	72 ± 8
Seated MAP, mmHg	89 ± 8
1 RM Bicep curl, kg	44.1 ± 13.6
20% 1 RM Bicep curl, kg	8.8 ± 2.8
60% LOP RL, mmHg	84 ± 10
60% LOP LL, mmHg	83 ± 9

Abbreviations: 1 RM, 1 repetition maximum; BMI, body mass index; DBP, diastolic blood pressure; HR, heart rate; LL, left leg; LOP, limb occlusion pressure; MAP, mean arterial pressure; RL, right leg; SBP, systolic blood pressure.

### 3.2 Cardiovascular outcomes

#### 3.2.1 Brachial blood pressure

No significant effects of condition (*F*(2,50) = 0.874; *p* = 0.424; *η* ^2^ *p* = 0.034; *β* = 0.192) or time (*F*(1,25) = 1.487; *p* = 0.234; *η* ^2^ *p* = 0.056; *β* = 0.216) were observed for systolic blood pressure (SBP). However, a significant interaction effect was found for diastolic blood pressure (DBP) (*F*(2,50) = 3.396; *p* = 0.041; *η* ^2^ *p* = 0.120; *β* = 0.613) (Table 2).

**TABLE 2:** Cardiovascular pre‐ to post‐assessment outcomes.

Variable	SC‐BFR		MC‐BFR		N‐BFR	
	Pre‐exercise	Post‐exercise	Pre‐exercise	Post‐exercise	Pre‐exercise	Post‐exercise
Supine SBP, mmHg	120 ± 10	120 ± 10	120 ± 12	122 ± 12	119 ± 11	120 ± 10
Supine DBP, mmHg	68 ± 9	62 ± 9^a^, ^b^	68 ± 10	66 ± 10	68 ± 8	62 ± 7^a^, ^b^
Supine MAP, mmHg	85 ± 8	81 ± 8^a^, ^b^	86 ± 9	84 ± 9	85 ± 8	81 ± 7^a^
Supine PP, mmHg	52 ± 11	58 ± 12^a^	52 ± 11	57 ± 12^a^	51 ± 10	59 ± 11^a^
Central SBP, mmHg	115 ± 14	115 ± 14	117 ± 17	115 ± 14	113 ± 14	113 ± 13
Central DBP, mmHg	68 ± 10	62 ± 9^a^	68 ± 10	66 ± 10	68 ± 8	62 ± 7^a^
Central MAP, mmHg	83 ± 9	79 ± 8^a^	84 ± 10	82 ± 10	83 ± 8	79 ± 7^a^
Central PP, mmHg	48 ± 16	53 ± 17^a^	49 ± 17	49 ± 14	46 ± 14	51 ± 15^a^
CR‐PWV, m/s	10.6 ± 2.4	10.4 ± 3.0	10.4 ± 2.3	10.3 ± 2.3	10.5 ± 2.5	10.2 ± 2.3
CF‐PWV, m/s	7.8 ± 1.7	7.6 ± 1.8	7.7 ± 1.4	8.2 ± 1.8	7.7 ± 1.3	7.8 ± 1.3

*Note*: Data are reported as mean and SD.

Abbreviations: CF, carotid‐femoral; CR, carotid‐radial; DBP, diastolic blood pressure; MAP, mean arterial pressure; PP, pulse pressure; PWV, pulse wave velocity; SBP, systolic blood pressure.

^a^ Significantly different from pre‐exercise.

^b^ Significantly lower than MC‐BFR.

Post‐exercise DBP was significantly reduced in the SC‐BFR condition (∆ = 6.192; 95% CI = 4.037 to 8.348; *p* < 0.001) and the N‐BFR condition (∆ = 5.846; 95% CI = 4.027 to 7.666; *p* < 0.001), while the MC‐BFR condition exhibited only a trend (∆ = 2.692; 95% CI = −0.176 to 5.516; *p* = 0.061). Furthermore, post‐exercise DBP values were significantly higher in the MC‐BFR condition compared to the SC‐BFR condition (∆ = 3.769; 95% CI = 0.385 to 7.153; *p* = 0.025) and the N‐BFR condition (∆ = 3.846; 95% CI = 0.735 to 6.957; *p* = 0.012).

A significant time effect was observed for mean arterial pressure (MAP) (*F*(1,25) = 20.779; *p* < 0.001; *η* ^2^ *p* = 0.454; *β* = 0.992). Post‐exercise MAP was significantly reduced in the SC‐BFR condition (∆ = 4.342; 95% CI = 2.355 to 6.329; *p* < 0.001) and the N‐BFR condition (∆ = 3.407; 95% CI = 1.716 to 5.098; *p* < 0.001), with no significant changes in the MC‐BFR condition (∆ = 1.140; 95% CI = −1.578 to 3.858; *p* = 0.396).

The two‐way ANOVA did not identify significant interaction effects (*p* = 0.084) or condition effects (*p* = 0.082) for MAP. However, post‐hoc analysis showed a significant post‐exercise difference between the MC‐BFR and SC‐BFR conditions (∆ = 3.356; 95% CI = 0.301 to 6.410; *p* = 0.028). Refer to Table 2.

#### 3.2.2 Central blood pressure

No significant effects of condition (*F*(2,50) = 0.749; *p* = 0.478; *η* ^2^ *p* = 0.029; *β* = 0.170) or time (*F*(1,25) = 0.743; *p* = 0.397; *η* ^2^ *p* = 0.029; *β* = 0.132) were observed for central systolic blood pressure (central SBP). However, a significant time effect was found for central diastolic blood pressure (central DBP) (*F*(1,25) = 41.589; *p* < 0.0001; *η* ^2^ *p* = 0.625; *β* = 1.000), along with a trend toward condition effects (*F*(2,50) = 2.950; *p* = 0.062; *η* ^2^ *p* = 0.106; *β* = 0.549) and interaction effects (*F*(2,50) = 2.863; *p* = 0.066; *η* ^2^ *p* = 0.103; *β* = 0.563).

Post‐exercise central DBP was significantly reduced in the SC‐BFR condition (∆ = 5.731; 95% CI = 3.402 to 8.060; *p* < 0.0001) and the N‐BFR condition (∆ = 5.846; 95% CI = 4.027 to 7.666; *p* < 0.0001), while no significant reduction was observed in the MC‐BFR condition (∆ = 2.615; 95% CI = −0.225 to 5.456; *p* = 0.070).

A significant time effect was also observed for MAP (*F*(1,25) = 23.109; *p* < 0.001; *η* ^2^ *p* = 0.480; *β* = 0.996). Post‐exercise central MAP was significantly reduced in the SC‐BFR condition (∆ = 4.060; 95% CI = 1.512 to 6.608; *p* = 0.003) and the N‐BFR condition (∆ = 4.073; 95% CI = 2.104 to 6.042; *p* < 0.0001), with no significant changes in the MC‐BFR condition (∆ = 2.510; 95% CI = −0.166 to 5.186; *p* = 0.065). Refer to Table 2.

#### 3.2.3 Pulse pressure

A significant time effect was observed for PP (*F*(1,25) = 65.513; *p* < 0.0001; *η* ^2^ *p* = 0.724; *β* = 1.000). Post‐exercise PP was significantly increased compared to baseline in the SC‐BFR condition (∆ = 5.538; 95% CI = 2.601 to 8.476; *p* = 0.001), MC‐BFR condition (∆ = 4.654; 95% CI = 2.135 to 7.172; *p* = 0.001), and N‐BFR condition (∆ = 7.308; 95% CI = 5.001 to 9.615; *p* < 0.0001).

A significant time effect was also observed for central pulse pressure (central PP) (*F*(1,25) = 6.173; *p* = 0.020; *η* ^2^ *p* = 0.198; *β* = 0.666). Post‐exercise central PP was significantly increased compared to baseline in the SC‐BFR condition (∆ = 5.000; 95% CI = 1.013 to 8.987; *p* = 0.016) and N‐BFR condition (∆ = 5.308; 95% CI = 1.500 to 9.115; *p* = 0.008), but no significant changes were observed in the MC‐BFR condition (∆ = 0.308; 95% CI = −4.624 to 5.240; *p* = 0.899). Refer to Table 2.

#### 3.2.4 Pulse wave velocity

For PWV‐CR, no interaction effect (W(2) = 0.143; *p* = 0.931), condition effect (W(2) = 0.097; *p* = 0.953), or time effect (W(1) = 0.839; *p* = 0.360) was observed. Similarly, for PWV‐CF, no interaction effect (W(2) = 2.545; *p* = 0.280), condition effect (W(2) = 0.423; *p* = 0.809), or time effect (W(1) = 0.492; *p* = 0.483) was reported. Refer to Table 2.

### 3.3 Performance

#### 3.3.1 Maximum number of repetitions

**FIGURE 5:**
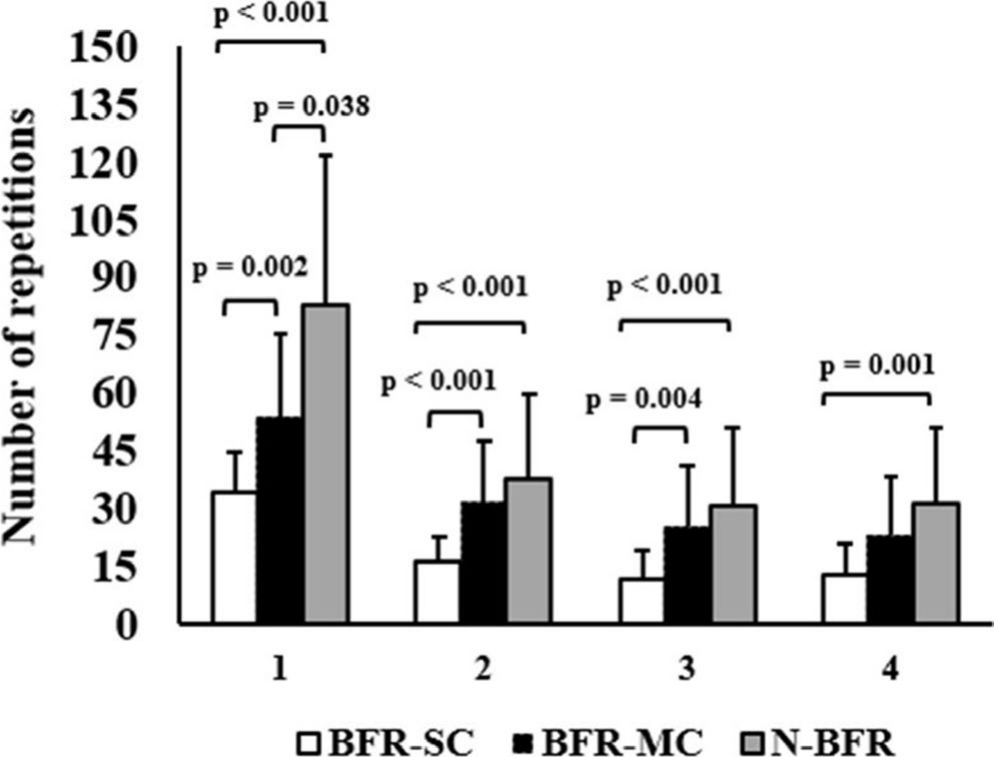
A maximal number of repetitions achieved during each trial is expressed in mean ± SD. SC‐BFR, single‐chambered BFR cuff; MC‐BFR, multi‐chambered BFR cuff; N‐BFR, no BFR condition.

A significant effect of condition was observed (W(2) = 55.577; *p* < 0.001). In the first set, the SC‐BFR protocol resulted in a significantly lower number of repetitions compared to MC‐BFR (∆ = −19.5; *p* = 0.002; 95% CI = −34.9 to −3.9) and N‐BFR (∆ = −48.7; *p* < 0.001; 95% CI = −74.7 to −22.8). A similar trend was seen in the second set, where SC‐BFR had fewer repetitions than MC‐BFR (∆ = −14.9; *p* < 0.001; 95% CI = −26.0 to −3.9) and N‐BFR (∆ = −21.4; *p* < 0.001; 95% CI = −36.0 to −6.5). In the third set, SC‐BFR continued to show significantly lower repetitions compared to MC‐BFR (∆ = −13.4; *p* = 0.004; 95% CI = −24.7 to −2.1) and N‐BFR (∆ = −19.08; *p* < 0.001; 95% CI = −32.9 to −5.2). However, by the fourth set, no significant difference was found between SC‐BFR and MC‐BFR (∆ = 10.3; *p* = 0.073; 95% CI = −21.0 to 0.34), although SC‐BFR still had significantly fewer repetitions compared to N‐BFR (∆ = −18.5; *p* = 0.001; 95% CI = −32.8 to −4.3) (Figure 5).

In the first set, MC‐BFR also showed a significantly lower number of repetitions compared to N‐BFR (∆ = −29.31; *p* = 0.038; 95% CI = −58 to −0.63). However, no significant differences were observed between MC‐BFR and N‐BFR in subsequent sets (*p* > 0.05). Table 3 depicts the per set repetition volumes for each condition.

**TABLE 3:** Average repetition performance per set and total in each condition.

Condition	Set 1 reps	Set 2 reps	Set 3 reps	Set 4 reps	Total reps
N‐BFR	82.9 ± 38.7	37.8 ± 22.1	31.0 ± 20.1	30.9 ± 19.9	131.9 ± 111.6
MC‐BFR	53.6 ± 21.5^a^	31.4 ± 15.9	25.3 ± 15.8	23.4 ± 14.9	96.6 ± 76.0^a^
SC‐BFR	34.1 ± 10.6^a^, ^b^	16.5 ± 6.1^a^, ^b^	11.5 ± 7.4^a^, ^b^	10.9 ± 8.3^a^	52.7 ± 40.2^a^, ^b^

*Note*: Data are reported as mean and SD.

Abbreviations: MC‐BFR, multi‐chambered BFR cuff; N‐BFR, no BFR condition; SC‐BFR, single‐chambered BFR cuff.

^a^ Significantly lower than N‐BFR.

^b^ Significantly lower than MC‐BFR.

#### 3.3.2 Perceptual responses and safety

For RPE, the Friedman ANOVA revealed differences between SC‐BFR and MC‐BFR (*p* = 0.025). No differences were reported between N‐BFR and MC‐BFR (*p* = 1.000) or SC‐BFR (*p* = 0.249). RPD was significantly higher in SC‐BFR compared to MC‐BFR and N‐BFR (*p* < 0.001), but there were no differences between MC‐BFR and N‐BFR (*p* = 1.0000). For the likelihood to perform, the Friedman test indicated no differences between the exercise conditions (*p* = 0.228) (Table 4).

**TABLE 4:** Perceptual post‐assessment outcomes.

Condition	Median	25–75th	Minimum–maximum	Mean rank
RPE				
SC‐BFR	9.5	8.75–10.0	5–10	2.40
MC‐BFR	9.0	7.75–9.25	6–10	1.67^a^
N‐BFR	9.0	8.0–10.0	6–10	1.92
RPD				
SC‐BFR	8.0	6.0–9.0	4–10	2.77
MC‐BFR	6.0	5.0–7.0	2–9	1.69^a^
N‐BFR	5.0	5.0–6.25	2–9	1.54^a^
Likelihood to perform again				
SC‐BFR	7.5	5.0–10.0	0–10	1.77
MC‐BFR	8.0	7.0–10.0	3–10	2.12
N‐BFR	9.0	8.0–10.0	2–10	2.12

Abbreviations: MC‐BFR, multi‐chambered BFR cuff; N‐BFR, no BFR condition; RPD, rating of perceived discomfort; RPE, rating of perceived exertion; SC‐BFR, single‐chambered BFR cuff.

^a^ Significantly lower than SC‐BFR.

No adverse events were reported in any condition during our trial, including in the familiarization session. For most participants, not being able to maintain the prescribed cadence was the most typical cause of volitional failure.

## 4 DISCUSSION

This study is the first to compare performance, perceptual responses, and cardiovascular outcomes during upper‐body exercise between single‐chambered (SC) and multi‐chambered (MC) bladder BFR cuff systems, alongside a low‐load no‐BFR (N‐BFR) condition. Our findings indicate that SC‐BFR and N‐BFR significantly reduced post‐exercise diastolic blood pressure (DBP) and mean arterial pressure (MAP), whereas MC‐BFR did not exhibit significant reductions. None of the conditions induced post‐exercise changes in pulse wave velocity (PWV) or central systolic blood pressure (SBP). In terms of performance, SC‐BFR (Delfi Personalized Tourniquet Device) significantly reduced repetitions across all sets compared to MC‐BFR (B Strong Cuff) and N‐BFR, with MC‐BFR performing similarly to N‐BFR after the first set. Regarding perceptual responses, MC‐BFR elicited lower ratings of perceived exertion (RPE) and discomfort (RPD) compared to SC‐BFR. SC‐BFR produced significantly higher RPD than both MC‐BFR and N‐BFR, with no differences between MC‐BFR and N‐BFR. Our hypothesis was partially supported, as PWV remained unchanged in all conditions, and performance was lower in SC‐BFR compared to the other conditions, with MC‐BFR resembling N‐BFR in later sets. However, contrary to our hypothesis, perceptual demands were lower in MC‐BFR, as it yielded significantly lower RPE scores compared to SC‐BFR and N‐BFR. These findings highlight the influence of bladder design on fatigue stimulus and perceptual responses during exercise, emphasizing the need to consider cuff design when refining clinical and practical BFR recommendations.

### 4.1 Cardiovascular

The analysis of blood pressure and vascular responses revealed distinct effects across conditions. Systolic blood pressure (SBP) showed no significant changes due to condition, time, or interaction. However, diastolic blood pressure (DBP) demonstrated a significant interaction effect (*p* = 0.041), with post‐exercise reductions observed in SC‐BFR and N‐BFR, while MC‐BFR displayed only a trend toward reduction while being significantly higher post‐exercise compared to N‐BFR and SC‐BFR. The post‐exercise reduction in DBP in SC‐BFR and N‐BFR exceeds the 5 mmHg threshold for clinical significance (Law et al., 2009), indicating a potentially therapeutic effect with chronic implementation, and in line with the findings of a prior study (Rolnick, De Queiros, Moghaddam, Marquette, et al., 2024b). While Rolnick et al. investigated the acute impact of autoregulation, they employed a similar resistance training protocol and found a small 4 mmHg elevation in post‐exercise SBP in the N‐BFR group only, which partially aligns with the SBP in the current study as no group displayed elevations. This may be due to the impact of upper‐body versus lower‐body exercise, although this is speculative. For DBP, all groups in Rolnick, De Queiros, Moghaddam, Marquette, et al. (2024b) displayed post‐exercise reductions ≥5 mmHg, which is in partial agreement as MC‐BFR displayed only a trend toward a reduction yet was still higher than N‐BFR and SC‐BFR. As both were acute studies that employed measurements 10 min post‐exercise in supine, future research should assess the immediate post‐exercise responses to make stronger conclusions regarding the peripheral blood pressure assessments. Other measures of cardiovascular responses include reductions in mean arterial pressure (MAP) in both SC‐BFR and N‐BFR without any appreciable changes in MC‐BFR. In addition, we found MAP was significantly lower in SC‐BFR compared to MC‐BFR, yet the difference was small (~3.5 mmHg). Central blood pressure findings mirrored these trends, with no significant changes observed in central SBP but a significant reduction in central DBP for SC‐BFR and N‐BFR, while MC‐BFR showed no significant reduction. We also observed pulse pressure (PP) increased significantly across all conditions for both peripheral and central measures, with the largest changes observed in N‐BFR. Notably, MC‐BFR showed no significant change in central PP. Possibly, the MC‐BFR condition, which displayed elevated DBP, likely had greater vascular resistance or reduced arterial compliance, which can maintain SBP but increases diastolic pressure (Homan et al., 2025). We speculate that in the N‐BFR and SC‐BFR conditions, with decreased DBP and increased PP, likely exhibit improved vascular compliance or vasodilation, leading to a wider pressure difference without altering SBP. Future research is needed to explore whether the acute observations made are practically significant in mitigating the risk of adverse responses in BFR exercise, including individuals with cardiovascular disease.

Last, pulse wave velocity (PWV) did not exhibit significant effects for interaction, condition, or time for CF‐PWV or CR‐PWV. This finding is in alignment with prior research in the upper body using a similar protocol (Rolnick, De Queiros, Moghaddam, Marquette, et al., 2024b) but conflicts with some data from other studies in the lower body investigating autoregulated pressures or MC‐BFR (Rolnick, de Queiros, Moghaddam, Peikon, et al., 2024; Rolnick, Licameli, Moghaddam, Marquette, et al., 2024). Currently, there is no accepted guideline on whether an acute increase in CF‐PWV is concerning. However, long‐term increases of +1 m/s in CF‐PWV have been associated with an increased risk of cardiovascular disease (Kim & Kim, 2019). The current findings indicate that exhaustive upper body bilateral single‐joint exercise with or without BFR does not impact PWV. Future research should investigate the responses in those with cardiovascular disease to assess whether the same observations are made, as that may have a significant impact on BFR use in clinical practice.

### 4.2 Performance

The results of our study reinforce previous findings that the type of bladder used in BFR resistance exercise significantly impacts acute exercise repetition performance. Specifically, when the SC‐BFR cuff was inflated to 60% of supine limb occlusion pressure (LOP) and compared to the MC‐BFR manufacturer's recommended application setting of 200 mmHg, the total repetitions completed across 4 sets to volitional muscular failure varied considerably. SC‐BFR resulted in approximately 60% fewer total repetitions than N‐BFR (~110 fewer repetitions) and approximately 45% fewer total repetitions than MC‐BFR (~61 fewer repetitions). In contrast, MC‐BFR completed approximately 27% fewer total repetitions than N‐BFR (~49 fewer repetitions). We also observed significant differences in the magnitude of the coefficient of variation (ϲ_ν%_) associated with the group‐level means, with the largest in the N‐BFR condition (ϲ_ν_ = 48.4%) followed by MC‐BFR (ϲ_ν_ = 40.4%) and then SC‐BFR (ϲ_ν_ = 36.9%). The reduced ϲ_ν_ in the SC‐BFR may indicate a more consistently applied stimulus compared to MC‐BFR, although more research is needed to determine the different participant characteristics and cuff placements that contribute to variations in exercise performance between conditions.

The largest per‐set repetition volume discrepancy occurred during Set 1, where SC‐BFR achieved 34 repetitions, MC‐BFR achieved 54 repetitions, and N‐BFR achieved 83 repetitions. This represents approximately 59% fewer repetitions (~49 fewer reps) for SC‐BFR compared to N‐BFR and approximately 36% fewer repetitions (~19 fewer reps) compared to MC‐BFR. Similarly, MC‐BFR completed approximately 35% fewer repetitions (~29 fewer reps) compared to N‐BFR. The reduction in repetitions persisted for SC‐BFR across all sets compared to N‐BFR and in Sets 2–3 when compared to MC‐BFR.

A prior study comparing repetition performance across 4 sets in the lower body between SC‐BFR, MC‐BFR, and N‐BFR using a similar methodology found that SC‐BFR resulted in significantly fewer total repetitions compared to the other conditions and that after Set 1, MC‐BFR performed similarly to N‐BFR (Rolnick, de Queiros, Moghaddam, Peikon, et al., 2024).

The findings of the current study largely align with the prior study, indicating that when inflated to the recommended pressure settings for each BFR cuff, total repetition volumes during BFR exercise are reduced with SC‐BFR compared to MC‐BFR. However, this study diverges in that the MC‐BFR condition produced significantly fewer repetitions than N‐BFR during upper body exercise, whereas in the prior lower body exercise study, both conditions performed similarly in total median number of repetitions.

The only other study to investigate the impact of repetition performance between cuffs of different bladder designs (Delfi Personalized Tourniquet Device and SmartTools SmartCuffs inflated to 50% LOP, and BStrong Cuffs inflated to 200 mmHg) during upper body exercise at 20% 1RM concluded that all cuffs reduced repetition performance similarly over two sets (Dancy et al., 2023). However, significant methodological limitations in the study limit the generalizability of its findings. Specifically, participants only performed two of the three cuff conditions, there was no no‐BFR control condition, and only two sets were performed instead of the four sets typically recommended in practice. Notably, the Delfi Personalized Tourniquet Device demonstrated the largest reduction in repetitions from Set 1 to Set 2 among all cuff conditions, suggesting that the potential impact of bladder design on repetition performance may have been underestimated. The current study underscores that this effect was likely missed in Dancy et al. (2023).

### 4.3 Perceptual responses and safety

The results of our study largely contradict our initial hypothesis, as MC‐BFR exhibited lower overall perceptual demands compared to SC‐BFR. Specifically, MC‐BFR demonstrated lower ratings of perceived exertion (RPE) than the other two conditions and lower ratings of perceived discomfort (RPD) compared to SC‐BFR, though similar to N‐BFR. However, despite the statistical significance observed, the absolute differences in RPE between conditions were relatively small and likely of limited practical significance. In contrast, the median and interquartile range (25%–75%) values for RPD may hold clinical relevance for BFR exercise—at least within the context of the protocol employed in our study.

Given that our protocol was designed to be maximal in intensity, we anticipated participants to report near‐maximal RPE scores, consistent with prior findings (Morishita et al., 2018). This expectation was confirmed, with 25%–75% percentile scores ranging from 7.75 to 10 across all conditions, indicating that participants experienced high levels of perceptual demand.

Regarding discomfort, MC‐BFR and N‐BFR elicited significantly lower RPD scores compared to SC‐BFR. However, the interquartile ranges of MC‐BFR and N‐BFR (5.0–7.0) suggest moderate to high levels of discomfort, aligning with existing literature on maximal‐intensity exercise, both with and without BFR (Rolnick, De Queiros, Moghaddam, Marquette, et al., 2024b; Rolnick, de Queiros, Moghaddam, Peikon, et al., 2024). Notably, SC‐BFR resulted in higher discomfort levels, with a median RPD score of 8.0 compared to 6.0 in MC‐BFR and 5.0 in N‐BFR, and a 25%–75% range of 6.0 to 9.0, indicating high to very high discomfort. These perceptual response patterns diverge from findings in a previous lower‐body study, which reported no significant influence of bladder design on perceptual responses during lower‐body exercise. This discrepancy may be attributed to the lower absolute pressure applied to the upper body in the current study (200 mmHg) compared to the 300 mmHg used for the lower body in the previous research (Rolnick, de Queiros, Moghaddam, Peikon, et al., 2024). However, this aligns with the manufacturer's recommended pressure settings, considering the multi‐chambered bladder cuff's limited occlusive capability (https://bstrong.training/pages/faq, 2025). Importantly, the elevated discomfort observed in the SC‐BFR condition may have clinical relevance to long‐term adherence (Rolnick et al., 2021), warranting further investigation.

Despite the differences in perceptual responses, no significant differences were observed in participants' willingness to repeat the exercise, suggesting that varying discomfort levels associated with different bladder designs had minimal influence on exercise adherence. Further research is necessary to determine whether these trends persist in clinical musculoskeletal populations, utilizing both the failure‐based protocol employed in this study and alternative non‐failure fixed repetition schemes.

Last, we did not observe adverse responses from any condition during our study. These findings are in line with some prior research (Rolnick, de Queiros, Moghaddam, Peikon, et al., 2024; Rolnick, Licameli, Moghaddam, Marquette, et al., 2024) but not all (Rolnick, De Queiros, Moghaddam, Marquette, et al., 2024b), despite the one adverse event reported in the latter being non‐BFR related. Nonetheless, it appears that in a mixed cohort of healthy, trained adults, BFR does not heighten the risk of adverse responses during exhaustive exercise bouts. More research is needed to understand factors that may be associated with increased risk of adverse responses to BFR exercise.

### 4.4 Limitations

This study is the first to investigate the impact of BFR bladder type on performance, perceptual responses, and cardiovascular and hemodynamic changes in the upper body using a repetition scheme that is frequently studied in research; however, it is not without limitations. First, the applied cuff pressures in the SC‐BFR and MC‐BFR conditions were not relativized to a consistent percentage of limb occlusion pressure (LOP). The inability to personalize pressure settings is an inherent feature of MC‐BFR cuffs (Rolnick, 2024; Rolnick et al., 2023), necessitating the use of manufacturer‐recommended settings. While this design limits direct comparisons of applied pressure, it reflects the real‐world application of the BFR cuff. Consequently, we implemented the manufacturer's recommended pressure setting for the MC‐BFR cuff (200 mmHg) (https://bstrong.training/pages/faq, 2025) and applied a moderate %LOP for the SC‐BFR condition in alignment with recent practical guidelines using single‐chambered systems (Patterson et al., 2019). Future research should explore methods to standardize pressures across different cuff designs to better isolate the influence of bladder type on acute BFR exercise responses. Additionally, incorporating supplementary measures, such as deoxygenated hemoglobin, metabolic stress markers including lactate and other contraction‐mediated molecules, may provide deeper insights into pressure‐dependent relationships. Second, our sample included resistance‐trained healthy males and females; however, the study was likely underpowered to detect sex differences. Third, although previous research suggests that different menstrual phases do not significantly affect arterial stiffness measures (Priest et al., 2018; Shenouda et al., 2018; Williams et al., 2020, 2024), their impact on perceptual responses remains unclear. Thus, caution is warranted when generalizing findings across populations. Fourth, the absence of a sham condition—where a cuff is applied but not inflated—precluded blinding between conditions. Future studies should consider incorporating a sham BFR condition to determine the extent to which observed effects are influenced by psychological factors. Fifth, this study was conducted in healthy, trained adults, and the results should not be extrapolated to clinical populations without a degree of caution. Last, the acute nature of this study does not allow for extrapolation into chronic usage. Longer‐term study designs are needed to explore trends and adaptations between cuffs.

## 5 CONCLUSIONS

This study aimed to assess the influence of BFR bladder design on acute performance, perceptual responses, and cardiovascular outcomes during bilateral upper‐body single‐joint resistance exercise. We demonstrate that SC‐BFR induces greater fatigue, reducing total repetitions performed compared to MC‐BFR and N‐BFR, while MC‐BFR elicits lower perceptual demands and discomfort similar to N‐BFR. Cardiovascular responses reveal that SC‐BFR and N‐BFR significantly reduce post‐exercise DBP and MAP, whereas MC‐BFR does not, indicating potential therapeutic implications. Despite these differences, no condition influenced CF‐PWV or central SBP, suggesting minimal impact on arterial stiffness. The study highlights the importance of cuff design considerations when implementing BFR and underscores the need for future research to explore long‐term adaptations, pressure standardization, and applications in clinical populations.

## AUTHOR CONTRIBUTIONS

NR conceived the project. TW performed data collection. NR, VSQ, and TW performed data analysis and wrote the first draft. VSQ and TW constructed the figures, and NR, VSQ, and TW constructed the tables. All authors participated in revising the manuscript and approved the final draft.

## FUNDING INFORMATION

No funding has been used for the present work.

## CONFLICT OF INTEREST STATEMENT

Nicholas Rolnick is the founder of THE BFR PROS, a BFR education company that provides BFR training workshops to fitness and rehabilitation professionals across the world using a variety of BFR devices. The other authors declare no potential or actual conflicts of interest. The results of the study are presented clearly, honestly, and without fabrication, falsification, or inappropriate data manipulation.

## ETHICS STATEMENT

The study was approved by the Salisbury University IRB Committee (Protocol #364). Every participant provided written informed consent for use of their anonymized data.

## Data Availability

Raw data is available upon reasonable request by contacting the corresponding author.
